# Neutrophil Lymphocyte Ratio as a Predictor of Stroke Severity in Type 2 Diabetes Mellitus: A Single-Center Study

**DOI:** 10.7759/cureus.51841

**Published:** 2024-01-08

**Authors:** Karan Pal Singh, Ramandeep Singh, Tanveer Singh, Sukhmandeep Kaur, Tinish Mittal, Meena Goyal, Pugazhendi Inban, Agaba Barnett James Musiime, Madiha D Haseeb, Aadil Khan

**Affiliations:** 1 Internal Medicine, Gandhi Medical College, Bhopal, Bhopal, IND; 2 College of Medicine, Punjab Institute of Medical Sciences (PIMS) Jalandhar, Jalandhar, IND; 3 Utilization Review, Good Samaritan Hospital, Bakersfield, USA; 4 Internal Medicine, Punjab Institute of Medical Sciences (PIMS) Jalandhar, Jalandhar, IND; 5 General Medicine, Government Medical College, Omandurar, Chennai, IND; 6 Internal Medicine, Royal College of General Practitioners (RCGP), London, GBR; 7 Neurology, Dow University of Health Sciences, Karachi, PAK; 8 Trauma Surgery, OSF St. Francis Medical Centre, University of Illinois Chicago, Chicago, USA; 9 Cardiology, University of Illinois Chicago, Chicago, USA; 10 Internal Medicine, Lala Lajpat Rai (LLR) Hospital, Kanpur, IND

**Keywords:** national institute of health stroke scale (nihss), “hba1c”, “stroke severity”, “ischemic stroke”, “type 2 diabetes mellitus”

## Abstract

Introduction: Type 2 diabetes mellitus (T2DM) is associated with various microvascular and macrovascular complications. Stroke, being a vascular complication, is associated with severe morbidity and mortality. Neutrophil lymphocyte ratio (NLR), a crude, inexpensive, and rather easily available modality to detect inflammation, has been utilized to find the extent of inflammation in type 2 diabetes mellitus patients. In this study, we find the effect of hemoglobin A1c (HbA1c) on NLR and the effect of NLR on stroke severity index.

Aims and objectives: This study aims to determine the use of the NLR in predicting stroke severity in a type 2 diabetes mellitus patient.

Materials and methods: This study is an observational cross-sectional study. A total of 400 patients were enrolled, all of whom had type 2 diabetes mellitus, with 200 of them diagnosed with an ischemic stroke. The National Institute of Health stroke scale (NIHSS) was used to standardize stroke severity and NLR was calculated from differential counts.

Results: The mean NLR for patients with type 2 diabetes mellitus was 3.87 ± 0.76 (mean ± SD), while for those with type 2 diabetes mellitus and stroke, it was 7.89 ± 1.29 (mean ± SD), with a statistically significant p-value < 0.001. Additionally, for every 1 unit increase in HbA1c, the NLR increased by 0.38 in type 2 diabetes mellitus patients and 0.86 in type 2 diabetes mellitus patients with stroke. Furthermore, each 1-unit increase in NLR corresponded to a rise of 0.80 in the stroke severity index.

Conclusion: The study shows a significant correlation between NLR in type 2 diabetes mellitus patients and stroke in type 2 diabetes mellitus patients. Also, it shows the significance of NLR in predicting stroke severity.

## Introduction

Urbanization and economic growth rapidity have increased the diabetes burden in various parts of the world due to many causes [1]. Among patients accessing advanced care, 4% had type 2 diabetes, with 57% of them facing complications, mainly cardiovascular issues. Direct medical costs totaled US $576 million, with 74% allocated to managing complications. Those with complications incurred double the costs compared to those without, emphasizing the significant financial impact of diabetes-related complications [2]. Diabetes ranks 7th leading cause in terms of human suffering considered in the Disability Adjusted Life Years (DALYs) [3]. A study by Chaveepojnkamjorn et al. on multivariable conditional logistic regression analysis found that hemoglobin A1c (HbA1c) levels of >8% were significantly associated with the development of ischemic stroke. Furthermore, higher HbA1c levels were associated with an increased risk of ischemic stroke [4]. Xue et al. found that a higher neutrophil-lymphocyte ratio (NLR) is associated with unfavorable short-term outcomes, higher stroke severity on admission, and recurrent ischemic strokes. A higher NLR was found to be associated with a higher risk of moderate-to-severe stroke severity at the time of admission. Also in a multivariable model, it was found that NLR was associated with unfavorable primary outcomes with adjustment done for the National Institute of Health stroke scale (NIHSS) score having odds ratio OR - 1.455, 95% CI of 1.083-1.956, p-value = 0.013 [5].

In a study, it was found that patients with hyperglycemia and higher NLR had >70% probability of having a mRS (modified Rankin score) >3 with p<0.001 and only 43% probability in patients with low NLR and hyperglycemia with p<0.001 [6]. Another Indian study done by Chittawar et al. demonstrated the correlation of NLR with diabetic nephropathy and retinopathy in the Indian population, showing the association of NLR with various other microvascular complications of type 2 diabetes mellitus (T2DM) [7]. Multiple studies have been done showing the relationship between NLR and stroke, whereas others describe the correlation between hyperglycemia and NLR with positive findings in both. It has also been seen that type 2 diabetes mellitus patients have a slightly raised NLR as compared to their healthy counterparts. However, only a few pieces of literature are available on the relationship between NLR and stroke in type 2 diabetes mellitus patients. The current research aims to explore the relationship between NLR (neutrophil-to-lymphocyte ratio) and the occurrence of stroke among patients diagnosed with type 2 diabetes mellitus.

## Materials and methods

Study design

An observational, cross-sectional study was conducted at the Department of Medicine, Hamidia Hospital, Gandhi Medical College, Bhopal. The study spanned from June 2018 to July 2020 and encompassed both outpatient (OPD) and inpatient cases from the medicine department.

Inclusion criteria

We included patients with type 2 diabetes mellitus who visited the medicine OPD and those who were admitted as inpatients due to ischemic stroke and also had type 2 diabetes mellitus.

Exclusion criteria

Patients with diabetes mellitus type 1 and those with various systemic disorders, including cardiovascular, kidney or liver disease, blood disorders, autoimmune conditions, malignancy, or poisoning, were excluded. In addition, we excluded individuals with infections, abnormal total leukocyte counts (TLCs), or those using specific medications.

Data collection

Data collection involved screening diagnosed type 2 diabetes mellitus patients and documenting their age, gender, medical history, including diabetes and hypertension, and any addiction habits such as alcohol intake, smoking, or tobacco chewing. Physical examinations covered general conditions, vital signs, BMI calculation, waist and hip measurements, and NIHSS score calculation for stroke patients.

Assessments and investigations

The assessment involved employing the NIHSS to evaluate stroke-related neurological deficits. Additionally, various blood investigations were conducted, including the complete blood count (CBC), kidney function test (KFT), liver function test (LFT), lipid profile, fasting blood sugar (FBS), postprandial blood sugar (PPBS), and HbA1c. These tests collectively provided insights into different aspects of health, including blood composition, kidney and liver function, lipid levels, and glucose control, enabling a comprehensive assessment of the patients' health status and potential risk factors related to their conditions. Metabolic and glycemic parameters, along with lipid profiles, were compared between the two groups.

Data management and analysis

Recorded data were coded and organized in MS Excel (Microsoft® Corp., Redmond, WA). Statistical analysis was performed using SPSS v23 (IBM Corp., Armonk, NY).

Ethical approval

The Institutional Ethics Committee of Gandhi Medical College, Bhopal, issued approval (No. 35601-B/MC/IEC/2018). All participants provided informed consent prior to their involvement in the study.

## Results

In this study, a total of 400 participants were enrolled, with 200 diagnosed with T2DM presenting in the Medicine Outpatient Department (OPD), and another 200 diagnosed with type 2 diabetes mellitus admitted as inpatients due to ischemic stroke. A comprehensive comparison between the two groups encompassed a range of variables, including age, gender, metabolic parameters, glycemic indicators, lipid profiles, and hematological factors, as shown in Table 1.

**Table 1:** Comparison of mean values of different variables in the case group (type 2 diabetes mellitus patients with stroke; n=200) and the control group (type 2 diabetes mellitus patients; n=200) with their p-values. *Significant at p<0.05, ^1^Wilcoxon-Mann-Whitney U test, ^2^chi-squared test. NLR: neutrophil-lymphocyte ratio, TLC: total lymphocyte count, HbA1c: hemoglobin A1c, FBS: fasting blood sugar, PPBS: postprandial blood sugar, W/H ratio: waist to hip ratio, BMI: body mass index, HDL: high-density lipoprotein, LDL: low-density lipoprotein.

Variables	Mean (case group) ± SD	Mean (control group) ± SD	p-value
NLR	7.89 ± 1.29	3.87 ± 0.76	<0.001^1^
TLC (/cu.mm)	8374.85 ± 1440.96	8390.95 ± 1393.83	0.880^1^
Stroke Severity Index	34.42 ± 3.48	NA	<0.001^1^
HbA1c (%)	10.60 ± 1.35	9.86 ± 1.86	<0.001^1^
FBS (mg/dL)	154.18 ± 19.99	166.22 ± 15.26	<0.001^1^
PPBS (mg/dL)	259.79 ± 49.21	267.17 ± 49.65	0.154^1^
Waist circumference (cm)	100.12 ± 2.54	99.60 ± 2.94	0.090^1^
Hip circumference (cm)	100.00 ± 2.56	98.68 ± 2.98	<0.001^1^
W/H ratio	1.00 ± 0.03	1.01 ± 0.04	0.054^1^
Height (cm)	1.65 ± 0.09	1.68 ± 0.07	0.001^1^
Weight (kg)	88.06 ± 9.94	89.80 ± 9.23	0.100^1^
BMI (kg/m^2^)	32.61 ± 4.81	32.08 ± 4.28	0.251^1^
Cholesterol (mg/dL)	198.78 ± 30.27	225.46 ± 25.28	<0.001^1^
Triglycerides (mg/dL)	170.43 ± 17.53	170.00 ± 17.74	0.822^1^
HDL (mg/dL)	59.32 ± 11.98	53.85 ± 9.43	<0.001^1^
LDL (mg/dL)	157.92 ± 21.72	140.80 ± 35.13	<0.001^1^

Demographic analysis revealed a significant difference in mean age between the T2DM group (49.19 ± 9.06 years, mean ± SD) and the T2DM with stroke group (56.62 ± 9.40 years, mean ± SD). Hematological variables showed no significant difference in total leukocyte count (TLC), but notable distinctions in absolute neutrophil count (ANC) and absolute lymphocyte count (ALC). The neutrophil-to-lymphocyte ratio (NLR) exhibited a significant difference, with mean NLRs of 7.89 ± 1.29 in the case group and 3.87 ± 0.76 in the control group. The stroke severity index in T2DM with stroke patients was 34.42 ± 3.48, as shown in Table 2.

**Table 2:** Comparison of mean values of various leukocyte counts in the case group (type 2 diabetes mellitus patients with stroke; n=200) and the control group (type 2 diabetes mellitus patients; n=200) with their w-values and p-values. The variables TLC, ANC, ALC, and NLR were not normally distributed in the two subgroups of the variable groups. Thus, non-parametric tests (Wilcoxon-Mann-Whitney U test) were used to make group comparisons. The test statistic for the Wilcoxon-signed rank is the w-value. TLC: total leukocyte counts, ANC: absolute neutrophil count, ALC: absolute lymphocyte count, NLR: neutrophil lymphocyte ratio.

Variables	Mean (case group) ± SD	Mean (control group) ± SD	W-value	p-value
TLC (/cu.mm)	8374.85 ± 1440.96	8390.95 ± 1393.83	19825.500	0.880
ANC (/cu.mm)	7118.03 ± 1244.14	6407.31 ± 1084.35	26225.500	<0.001
ALC (/cu.mm)	921.24 ± 199.04	1706.14 ± 390.91	1154.500	<0.001
NLR	7.89 ± 1.29	3.87 ± 0.76	39955.000	<0.001

**Figure 1:**
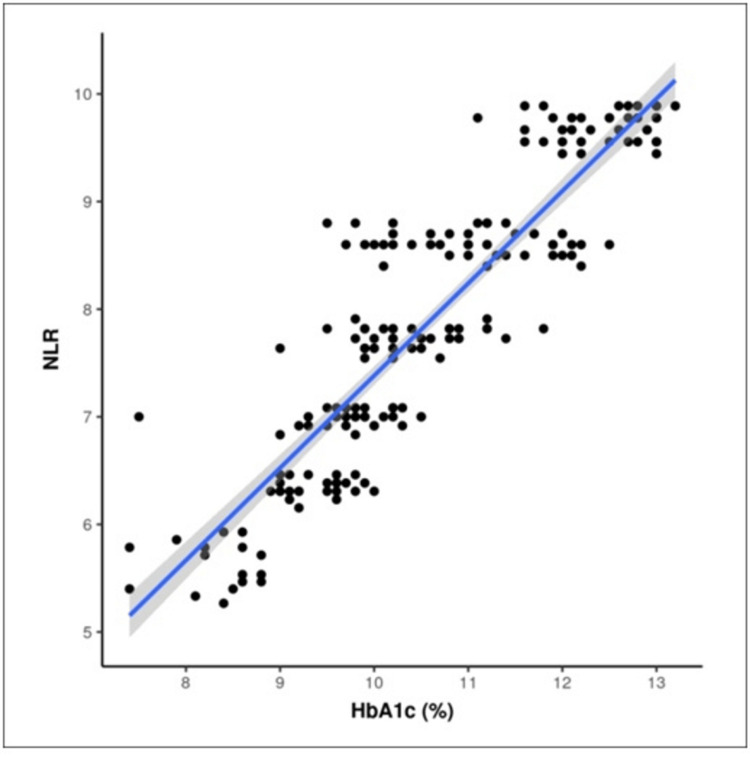
Scatter figure showing a correlation between HbA1c (%) and NLR in the case group. The X-axis represents HbA1c levels, and the Y-axis represents neutrophil-to-lymphocyte ratio. Each data point reflects the relationship between HbA1c and NLR. The analysis indicates that for every 1.0% increase in HbA1c, there is a corresponding increase in NLR by 0.86 (p-value < 0.001).

**Figure 2:**
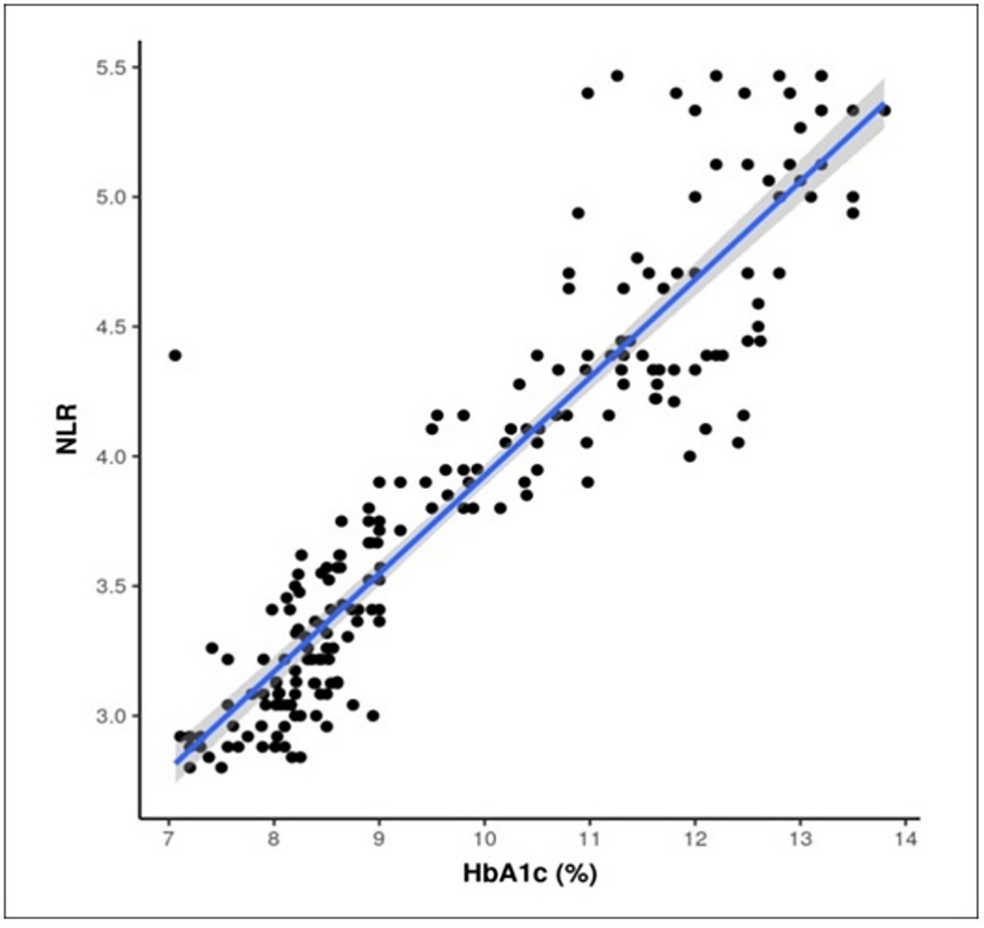
Scatter figure showing a correlation between HbA1c (%) and NLR in the control group. The X-axis represents HbA1c levels, and the Y-axis represents neutrophil-to-lymphocyte ratio. Each data point illustrates the relationship between HbA1c and NLR. The analysis reveals that for every 1.0% increase in HbA1c, there is a corresponding increase in NLR by 0.38 (p-value < 0.001).

Non-parametric tests, specifically Spearman correlation, were employed to investigate the relationship between NLR and HbA1c. Scatter plot diagrams were constructed for both the case and control groups, revealing a significant correlation between the two variables, as detailed in Table 3 and visually represented in Figures 1-2. The analyses indicated that for every 1.0% increase in HbA1c, NLR increased by 0.86 and 0.38 in the case and control groups, respectively, with a highly significant p-value of less than 0.001. These findings underscore a robust and consistent correlation between glycemic control, reflected in HbA1c levels, and inflammatory markers, as measured by NLR, in both patient groups.

**Table 3:** Spearman correlation with p-values between the neutrophil-lymphocyte ratio and hemoglobin A1c in the case group (type 2 diabetes mellitus patients with stroke; n=200) and the control group (type 2 diabetes mellitus patients; n=200). *Significant at p-value <0.05.

Correlation between NLR and HbA1c	Spearman correlation coefficient	P-value
Case group	0.890	<0.001
Control group	0.920	<0.001

**Figure 3:**
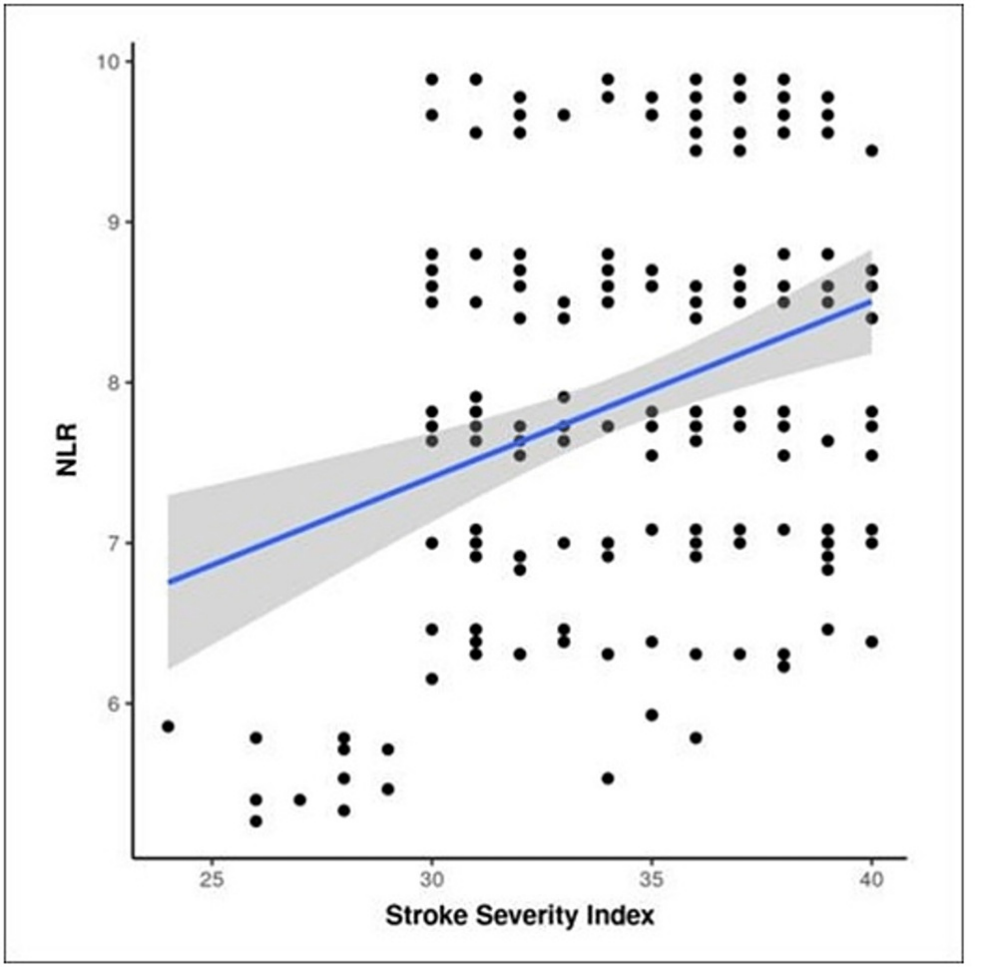
Scatter figure showing a correlation between stroke severity index and NLR. The X-axis represents stroke severity index, and the Y-axis represents neutrophil-to-lymphocyte ratio. Each data point illustrates the relationship between stroke severity index and NLR. The analysis indicates that for every 1 unit increase in NLR, there is a corresponding 0.80 increase in stroke severity index (Spearman’s rank correlation coefficient, rho=0.23, p-value = 0.001).

Non-parametric tests, specifically Spearman correlation, were employed to assess the relationship between NLR and stroke severity index. The analysis revealed a weakly positive yet statistically significant correlation. The findings indicated that for every 1 unit increase in NLR, there was a corresponding 0.80 increase in the stroke severity index of the patients. The Spearman's rank correlation coefficient (rho) for this association was 0.23, signifying the strength of the correlation, and the p-value was found to be 0.001, demonstrating the statistical significance of the observed relationship. These results are succinctly presented in Figure 3, providing a clear visual representation of the correlation between NLR and stroke severity index in the study cohort.

## Discussion

In 2015, the International Diabetes Federation (IDF) estimated that 1 in 11 adults (415 million adults, aged between 20 and 79 years) had diabetes mellitus worldwide [8]. With the increasing trend of diabetes, there is an increased rate of complications related to diabetes mellitus. HbA1c is a good test to diagnose diabetes mellitus, which has chronic hyperglycemia and gradually progresses to its various chronic complications [9].

The neutrophil-to-lymphocyte ratio, which is calculated by dividing the ANC by the ALC, which is routinely done in clinics, was found to be very reliable in comparison to the CBC to detect subclinical inflammation in both non-cardiac and cardiac diseases. There have been many studies showing the fact that NLR is not only a marker of inflammation but also has the potential for diagnosis and determining the prognosis of atherosclerotic vascular diseases, including diabetes mellitus [10]. Diabetes is an independent risk factor for ischemic-type stroke and has both higher mortality and morbidity [11]. In the present cross-sectional study, we have taken a total of 400 participants, out of which the control group has 200 participants who have been diagnosed with type 2 diabetes mellitus, and the cases group has 200 participants who were diagnosed with type 2 diabetes mellitus and have now presented with an ischemic stroke.

A comparison of lipid profiles showed significant differences in total cholesterol, HDL, and LDL between the case and control groups. Interestingly, triglyceride levels did not significantly differ, aligning with certain previous studies. A study by Patil and Raghuwanshi done in the same center as ours determined that total cholesterol, serum triglycerides, and LDL levels were risk factors for ischemic stroke, whereas the study could not establish a significant relationship for HDL levels in predicting the stroke, although this could be due to the small sample size of only n=50 people in the study [12]. In another study by Rhoads and Feinleib, there was no association found between serum triglyceride levels and the causation of stroke, which is similar to our study but in most large studies there was a positive correlation between them [13].

The glycemic control parameters, including HbA1c, FBS, and PPBS, were assessed in both the case and control groups. Notably, HbA1c levels demonstrated a significant difference between the two groups, suggesting its potential as a critical marker for ischemic stroke risk in diabetic patients. While FBS exhibited a significant difference between the case and control groups, PPBS did not show a notable distinction. This implies that fasting blood sugar levels might have a more pronounced impact on the risk of ischemic stroke in diabetic patients compared to postprandial levels. In another large study by Shen et al., it was found that HbA1C levels formed a U-shaped relationship with the incidence of stroke in a diabetes mellitus patient, which means that both poor and excellent control of hyperglycemia increase the incidence of ischemic stroke [14]. It is seen that at the HbA1C level of 7.5 ± 0.25%, there was a minimum risk of mortality from ischemic stroke in a diabetic patient [15]. Although this kind of U-shaped relationship is not present in our results, the difference in the sample size and ethnic variance may be the cause of the same. The mean ± standard deviation (SD) of NLR in the case group was 7.89 ± 1.29 and in the control group, it was 3.87 ± 0.76. The median of NLR in the case group was 7.82 and in the control group, it was 3.78. The NLR in the case group ranged from 5.27 to 9.89 and in the control group, it ranged from 2.8 to 5.47. A significant difference was found between the two groups in terms of NLR (p-value < 0.001), with the median NLR being highest in the case group. In another study by Hussain et al., there were three groups of patients based on HbA1c: A (<7%), B (7-9%), and C (>9%), and NLR was observed in all three, which came to be 2.0 ± 0.5, 2.7 ± 1.0 and 4.3 ± 2.8, respectively, with p < 0.001 [16].

The scatterplot depicting the correlation between stroke severity index and NLR in the cases group shows that there is a weak positive correlation between stroke severity index and NLR, although this correlation was found to be statistically significant (rho = 0.23, p-value = 0.001) and it was determined that for every 1 unit increase in NLR, the stroke severity index increases by 0.80 units. In conclusion, it can be stated that in diabetic patients, NLR is a tool that is inexpensive and readily available and has a strong association with patients with type 2 diabetes mellitus and patients with type 2 diabetes mellitus developing stroke. Also, it was found that as the HbA1C level increases (poor glycemic control), the NLR rises further, and with an increase in the NLR, the stroke severity index increases further. A prospective study is needed to establish a causal association between NLR and diabetes in diabetic patients.

It is important to acknowledge several limitations in this study. The cross-sectional design hinders the establishment of causal relationships, warranting the need for prospective investigations. The relatively small sample size of 400 participants, the potential selection bias associated with including individuals with a history of ischemic stroke, and the single-center nature of the study limit the generalizability of the findings. These limitations underscore the importance of cautious interpretation and emphasize the need for future research to address these constraints and provide more conclusive insights into the relationships observed in this study.

## Conclusions

Neutrophil-to-lymphocyte ratio emerges as a cost-effective and easily accessible tool in diabetic patients, showcasing a robust link with both T2DM and the occurrence of strokes. Moreover, the observed elevation in NLR aligns with higher HbA1c levels, indicating aggravated poor glycemic control, and corresponds to an intensified stroke severity index. However, establishing a cause-and-effect relationship necessitates a prospective study, essential for clarifying NLR's role in diabetic patients' complications and underscoring the imperative need for further research to validate these associations conclusively.
